# The ketogenic diet increases Neuregulin 1 expression via elevating histone acetylation and its anti-seizure effect requires ErbB4 kinase activity

**DOI:** 10.1186/s13578-021-00611-7

**Published:** 2021-05-21

**Authors:** Jin Wang, Jie Huang, Shan Yao, Jia-Hui Wu, Hui-Bin Li, Feng Gao, Ying Wang, Guo-Bin Huang, Qiang-Long You, Jianhua Li, Xiaohui Chen, Xiang-Dong Sun

**Affiliations:** 1 Emergency Department, Institute of Neuroscience, Department of Neurology of the Second Affiliated Hospital of Guangzhou Medical University, Key Laboratory of Neurogenetics and Channelopathies of Guangdong Province and the Ministry of Education of China, Guangzhou, 510260 China; 2 Department of Physiology, School of Medicine, Jinan University, Guangzhou, 510632 China; 3 Department of Pathology, Guangdong Women and Children Hospital, Guangzhou, 511400 China; 4 Key Laboratory of Protein Modification and Degradation, School of Basic Medical Science, Affiliated Cancer Hospital & Institute of Guangzhou Medical University, Guangzhou, China

**Keywords:** Neuregulin 1, Ketogenic diet, Acetylation, ErbB4 kinase activity, Epilepsy

## Abstract

**Background:**

The ketogenic diet (KD)has been considered an effective treatment for epilepsy, whereas its underlying mechanisms remain obscure. We have previously reported that the KD feeding increased Neuregulin 1 (NRG1) expression in the hippocampus; disruption of NRG1 signaling by genetically deleting its receptor-ErbB4 abolished KDs effects on inhibitory synaptic activity and seizures. However, it is still unclear about the mechanisms underlying the effect of KD on NRG1 expression and whether the effects of KD require ErbB4 kinase activity.

**Methods:**

The effects of the KD on NRG1 expression were assessed via western blotting and real-time PCR. Acetylation level at the *Nrg1* promoter locus was examined using the chromatin immunoprecipitation technique. Kainic acid (KA)-induced acute seizure model was utilized to examine the effects of KD and histone deacetylase inhibitor-TSA on seizures. Synaptic activities in the hippocampus were recorded with the technique of electrophysiology. The obligatory role of ErbB4 kinase activity in KDs effects on seizures and inhibitory synaptic activity was evaluated by using ErbB kinase antagonist and transgenic mouse-T796G.

**Results:**

We report that KD specifically increases Type I NRG1 expression in the hippocampus. Using the chromatin immunoprecipitation technique, we observe increased acetylated-histone occupancy at the *Nrg1* promoter locus of KD-fed mice. Treatment of TSA dramatically elevates NRG1 expression and diminishes the difference between the effects of the control diet (CD) and KD. These data indicate that KD increases NRG1 expression via up-regulating histone acetylation. Moreover, both pharmacological and genetic inhibitions of ErbB4 kinase activity significantly block the KDs effects on inhibitory synaptic activity and seizure, suggesting an essential role of ErbB4 kinase activity.

**Conclusion:**

These results strengthen our understanding of the role of NRG1/ErbB4 signaling in KD and shed light on novel therapeutic interventions for epilepsy.

**Supplementary Information:**

The online version contains supplementary material available at 10.1186/s13578-021-00611-7.

## Background

Epilepsy is one of the most common neurological disorders that affects over 1% of people worldwide [1]. Although tremendous progress has been achieved during the last decades, the underlying pathological mechanisms are still elusive. In the clinic, approximately one-third of the patients exhibit resistance to drug treatments [2], which expresses the urge to identify novel molecular targets for epilepsy to develop highly potent therapeutic strategies.

The ketogenic diet (KD) is a low-carbohydrate, moderate-protein, and high-fat diet known to induce a sustained ketotic state by producing heightened ketone bodies. Since the 1920s, the KD has been utilized to treat refractory epilepsy in the clinic [3]. However, its application has mainly been restricted because of dietary compliance and adverse side effects [4]. Understanding the critical cellular and molecular mechanisms underlying the therapeutic effects of the KD will provide insights into mechanisms of epilepsy and facilitate developing effective pharmacological strategies.

Neuregulin 1 (NRG1) belongs to a trophic-factor family and contains an epidermal growth factor (EGF) domain [5]. Due to alternative splicing and multiple promoters, the *Nrg1* gene produces more than 30 different isoforms comprising six types of protein: I-VI, in which the I, II, and III are the primary types [6]. A distinct amino-terminal sequence differentiates various NRG1 protein types, whose expression patterns and levels are diverse in the brain [7, 8]. Located in the membrane-proximal region of the extracellular domain, the EGF-like domain is sufficient to activate its receptors-ErbB tyrosine kinase, including EbrB4 [9]. By stimulating the ErbB4 receptor, NRG1 plays essential roles in neural development, GABAergic circuits assembly, neurotransmission, and synaptic plasticity [10]. In our previous work, we reported that NRG1 expression was dramatically elevated by KD treatment. Disruption of NRG1 signaling by genetically deleting *ErbB4* diminished the KDs effects on GABAergic synaptic transmission and seizures [11]. These data suggest a critical role of NRG1/ErbB4 signaling in mediating KDs effects on GABAergic transmission and seizure. However, the mechanisms underlying the regulation of NRG1 expression by KD are still not understood.

Besides, evidence from in-vitro experiments demonstrated that both full-length and kinase-dead mutant of ErbB4 construct promoted presynaptic maturation [12], and type III NRG1 functioned in dendritic development, which was independent of ErbB4 kinase activity [13]. Moreover, NRG2 was reported to reduce synaptic GABA receptor-mediated currents, which were not blocked by ErbB kinase antagonist [14]. These studies suggest that NRG1/ErbB4 signaling may function independently of ErbB4 kinase activity. Nevertheless, whether the kinase activity of ErbB4 is needed in the anti-seizure effects of the KD is waiting to be elucidated.

In the present study, we find that KD feeding selectively increases type I NRG1 expression in the hippocampus. Using the chromatin immunoprecipitation (CHIP) technique, we observe increased acetylated-histone occupancy at the *Nrg1* promoter locus of KD-fed mice. Treatment of TSA, a broad-spectrum histone deacetylase (HDAC) inhibitor, dramatically elevates NRG1 expression in the hippocampus and diminishes the difference between the control diet (CD) and KD groups, indicating that KD increases NRG1 expression via up-regulating histone acetylation. Furthermore, both pharmacological and genetic inhibitions of ErbB4 kinase activity significantly block the KDs effects on inhibitory synaptic activity and seizure, suggesting an essential role of ErbB4 kinase activity. These results strengthen our understanding of the role of NRG1/ErbB4 signaling in KD.

## Result

### Type I NRG1 expression is specifically increased in the hippocampus by KD treatment

**Fig. 1:**
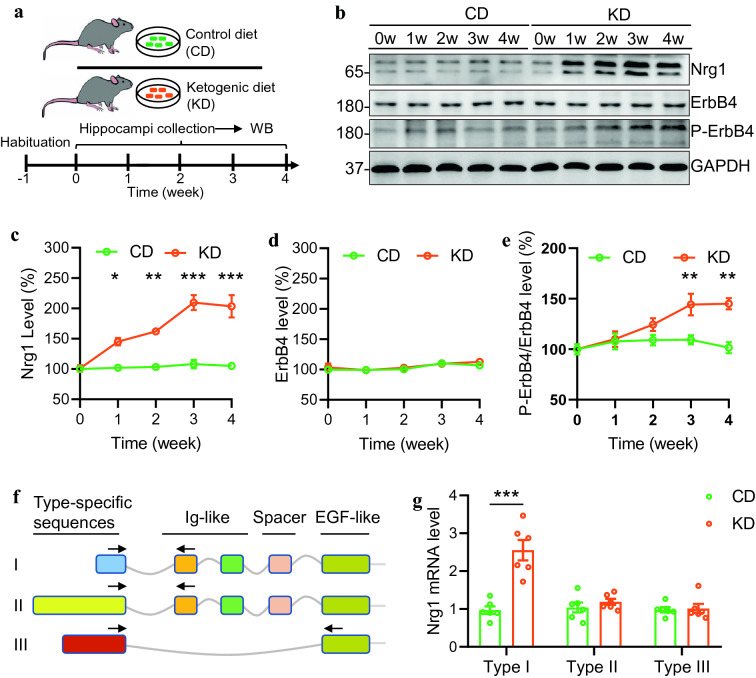
KD treatment specifically increases type I NRG1 expression in the hippocampus. **A** Experimental design. After 1 week of habituation, male adult mice were divided into two groups, fed with a control diet (CD) or KD for different time as indicated. Hippocampal tissues were collected and subjected to western blotting. **B** increased expression of NRG1 and phosphorylated ErbB4 (P-ErbB4) proteins in the hippocampus of mice fed with KD. Shown were representative blots. GAPDH served as the loading control. **C** Quantitative analysis of NRG1 expression in D. One-way ANOVA with sidaks multiple comparison tests. For CD group, F_(4,10)_=0.5538, *P*=0.7011; For KD group, F_(4,10)_=17.79, *P*=0.0002. 1 week vs 0 week, P=0.0459; 2 week vs 0 week, *P*=0.0073; 3 week vs 0 week, *P*=0.0001; 4 week vs 0 week, *P*=0.0002. **D** Quantitative analysis of ErbB4 expression in D. One-way ANOVA with sidaks multiple comparison tests. For CD group, F_(4,10)_=2.662, *P*=0.0954; For KD group, F_(4,10)_=2.922, *P*=0.077. **E** Quantitative analysis of P-ErbB4/ErbB4 levels in D. One-way ANOVA with sidaks multiple comparison tests. For CD group, F_(4,10)_=0.5962, *P*=0.6737; For KD group, F_(4,10)_=7.929, *P*=0.0038. 1 week vs 0 week, *P*=0.8158; 2 week vs 0 week, P=0.1382; 3 week vs 0 week, *P*=0.0055; 4 week vs 0 week, *P*=0.0049. **F** Diagram of *Nrg1* gene structure. Type-specific forward primers (indicated by arrows) for each type were located in unique exons, whereas reverse primers were located in either Ig or EGF domain. **G** Increased type I *Nrg1* mRNA level in the hippocampus of KD-fed mice. Two-way ANOVA with sidaks multiple comparison tests. F_(1,30)_=24.89, *P*<0.0001; For type I, *P*<0.0001; for type II, *P*=0.8537; for type III, *P*=0.9973. * indicates *p*<0.05; ** indicates *p*<0.01; *** indicates *p*<0.001

The adult C57/B6 mice were treated with KD or its control diet (CD). Three weeks later, we found that the serum level of -hydroxybutyrate (a major form of ketone) was dramatically increased in KD-fed mice (Additional file 1: Fig. S1A). Besides, the serum level of glucose was significantly decreased (Additional file 1: fig. S1B). These results indicate that the KD treatment can faithfully induce a state of high level of ketone bodies in mice. We then attempted to characterize the protein expression of NRG1 and ErbB4 in the hippocampus from CD or KD-fed mice (Fig. 1A). While the CD treatment exhibited little effect on NRG1 expression in the hippocampus, the NRG1 level was gradually increased in 1 week and peaked at 3 weeks of the KD treatment (Fig. 1B, C). However, the ErbB4 level was not altered in the hippocampus throughout the KD or CD treatment (Fig. 1B and D). Accordingly, the phosphorylated-ErbB4 (P-ErbB4) level was elevated after 3 weeks of KD treatment (Fig. 1B and E). These observations suggest that NRG1 rather than ErbB4 expression is dynamically regulated by the KD treatment.

It has been reported that multiple types of NRG1 exist in the mammalian brains, of which type I, II, and III NRG1 are the primary isoforms [8, 15, 16]. Types I and II rather than III have an Ig domain before the EGF domain (Fig. 1F). To examine which types are regulated by the KD treatment, we performed RT-qPCR to characterize the levels of various NRG1 isoforms in the hippocampus. We designed forward primers based on type-specific sequences and reverse primers against either the Ig (for types I, II) or EGF domain (for types III). We found that while type I *Nrg1* mRNA level was drastically increased in the hippocampus of KD-fed mice than in CD-fed mice, the levels of type II and type III *Nrg1* mRNA were not different between the CD and KD group (Fig. 1G). These results demonstrate a specific regulation of type I *Nrg1* expression by the KD treatment.

### KD increases NRG1 expression via histone acetylation

**Fig. 2:**
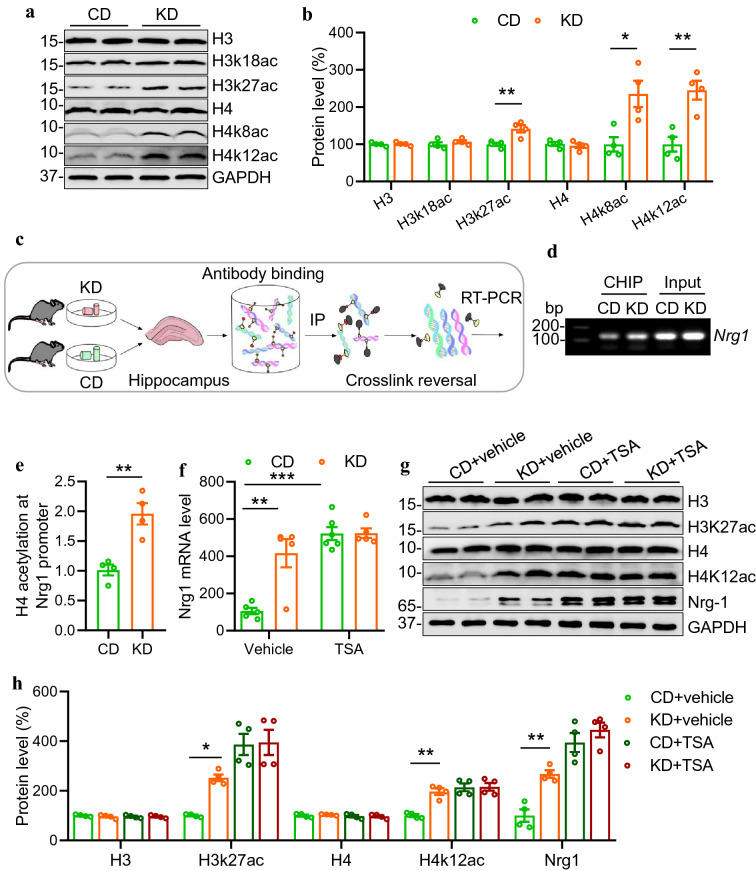
KD increases NRG1 expression via histone acetylation. **A** Representative Western blots showing H3, H4 proteins and their acetylation levels in the hippocampus of CD or KD-fed mice. GAPDH serves as a loading control. **B** Quantitative analysis of data in A. n=4 mice per group. Students *t* test, for H3, t_(6)_=0.2343, *P*=0.8225; for H3k18ac, t_(6)_=0.9896, *P*=0.3606; for H3k27ac, t_(6)_=3.816, *P*=0.0088; for H4, t_(6)_=0.6188, *P*=0.5588; for H4k8ac, t_(6)_=3.378, *P*=0.0149; for H4k12ac, t_(6)_=4.479, *P*=0.0042. **C** Hippocampal samples from CD and KD groups were subjected to Chromatin Immunoprecipitation (CHIP). Shown was the CHIP procedure workflow. **D** Agarose gel electrophoresis of CHIP-qPCR products. **E** Increased level of H4 acetylation at *Nrg1* promoter in KD-fed mice. n=4 mice per group. Students *t* test, t(6)=4.698, *P*=0.0033. **F** TSA treatment increased hippocampal *Nrg1* mRNA level and diminished the difference between CD and KD groups. n=5 mice per group except that n=6 mice for CD+TSA group. Two-way ANOVA with sidaks multiple comparison tests. F_(1,17)_=12.71, P=0.0024; CD+vehicle vs KD+vehicle, P=0.0008; CD+vehicle vs CD+TSA, *P*<0.0001; CD+TSA vs KD+TSA, *P*>0.9999. **G** NRG1 level in the hippocampus was increased by TSA treatment. Shown were representative western blot images. GAPDH serves as a loading control. **H** Quantitative analysis of western blot data in G. One-way ANOVA, for H3, F_(3,12)_=0.4619, *P*=0.7141; for H3K27ac, F_(3,12)_=15.9, *P*=0.0002. CD+vehicle vs KD+vehicle, P=0.0469; CD+vehicle vs CD+TSA, *P*=0.0004; CD+TSA vs KD+TSA, *P*>0.9999; for H4, F_(3,12)_=0.5403, P=0.6637; for H4K12ac, F_(3,12)_=17.86, *P*=0.0001. CD+vehicle vs KD+vehicle, *P*=0.0012; CD+vehicle vs CD+TSA, P=0.0003; CD+TSA vs KD+TSA, P>0.9999; For NRG1, F_(3,12)_=29.81, *P*<0.0001. CD+vehicle vs KD+vehicle, *P*=0.0072; CD+vehicle vs CD+TSA, *P*<0.0001; CD+TSA vs KD+TSA, P=0.7846. * indicates *p*<0.05; ** indicates *p*<0.01; *** indicates *p*<0.001

We then explored the underlying mechanisms through which KD treatment increases NRG1 expression. It has been reported that ketone body--hydroxybutyrate is a modulator of histone acetylation [17], which serves as a critical epigenetic mechanism underlying the regulation of gene expression [18]. To determine whether KD affects NRG1 expression through histone acetylation, we investigated the effects of KD on histone acetylation levels in the hippocampus by western blotting. Compared with mice fed with the CD, KD-fed mice exhibited increased levels of histone H3 acetylation at 27 lysine residues, as well as histone H4 at both 8 and 12 lysine residues. In contrast, the levels of total histone H3 or H4 proteins were not changed (Fig. 2A, B). These data suggest that KD treatment enhances histone acetylation in the hippocampus. To directly examine whether KD treatment regulates histone acetylation level at *Nrg1* promoter locus, we performed a CHIP assay using acetylated H3 and H4 antibodies as baits and a specific primer pair targeting the type I *Nrg1* promoter locus (Fig. 2C). We observed significantly increased occupancy of both acetylated H3 and H4 histones at the *Nrg1* promoter locus in KD-fed mice compared with those in CD-fed mice (Fig. 2D and E; Additional file 1: Fig. S2A, B), suggesting a potential effect of histone acetylation on NRG1 expression.

To further test the involvement of histone acetylation in the regulation of NRG1 expression, we injected C57/B6 mice with TSA (5 mg/kg body weight, i.p.), a broad-spectrum HDAC inhibitor [19]. TSA treatment dramatically increased the acetylation levels of H3 and H4 (Additional file 1: Fig. S1C, D), indicating a positive effect of TSA on acetylation, consistent with previous reports [20, 21]. We found that while KD treatment dramatically increased *Nrg1* mRNA level in the hippocampus compared with that in CD-fed mice, TSA injection elevated the *Nrg1* mRNA level in the CD-fed mice to a similar level of that in KD-fed mice injected with TSA (Fig. 2F). Furthermore, western blotting results demonstrated that TSA treatment diminished the difference of acetylated H3 and H4 protein levels between CD and KD groups. Remarkably, TSA treatment also diminished the difference of NRG1 protein level between the CD and KD group (Fig. 2G, H). In contrast, the pre-treatment of ITSA, an antagonist of TSA, blocked TSA's effect on NRG1 expression (Additional file 1: Fig. S2E, F). These observations provide strong evidence that KD promotes NRG1 expression via histone acetylation.

### KD promotes inhibitory synaptic activity and suppresses seizures via histone acetylation

**Fig. 3:**
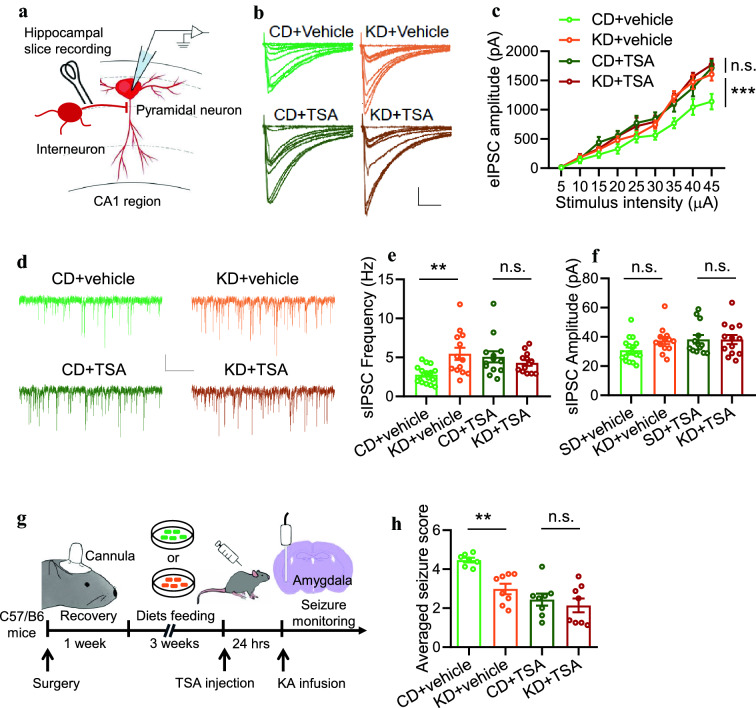
KD promotes inhibitory synaptic activity and suppresses seizures via histone acetylation. **A** Diagram of hippocampal slice recording. Pyramidal neurons in the CA1 region were clamped in whole-cell configuration. Evoked postsynaptic currents were recorded under stimulation by using a concentric bipolar electrode. **B** Representative eIPSC traces from pyramidal neurons in the CA1 region. Scale bars, 100 ms and 1000 pA. **C** TSA treatment (i.p., 5 mg/kg body weight) diminished the effect of KD on eIPSC amplitudes. n=16 neurons from 4 mice (CD+vehicle); n=21 neurons from 4 mice (KD+vehicel); n=16 neurons from 4 mice (CD+TSA); n=16 neurons from 4 mice (KD+TSA). Two-way ANOVA, CD+Vehicle vs KD+Vehicle, F_(1,315)_=28.27, *P*<0.0001; CD+TSA vs KD+TSA, F_(1,270)_=0.0771, *P*=0.7815. **D** Representative spontaneous inhibitory postsynaptic current (sIPSC) traces. Scale bars, 2 s and 20 pA. **E** TSA treatment diminished the effect of KD on sIPSC frequency. n=17 neurons from 4 mice (CD+vehicle); n=13 neurons from 4 mice (KD+vehicle); n=13 neurons from 4 mice (CD+TSA); n=13 neurons from 4 mice (KD+TSA). One-way ANOVA, F_(3,52)_=5.242, *P*=0.0031. CD+vehicle vs KD+vehicle, *P*=0.0044; CD+TSA vs KD+TSA, *P*=0.9099. **F** TSA treatment exhibited little effect on sIPSC amplitude. n=17 neurons from 4 mice (CD+vehicle); n=13 neurons from 4 mice (KD+vehicle); n=13 neurons from 4 mice (CD+TSA); n=13 neurons from 4 mice (KD+TSA). One-way ANOVA, F_(3,52)_=2.211, P=0.0978. **G** Schematic of experimental design. After 3 weeks of diet feeding, C57 mice were injected with TSA one day before KA infusion to induce seizures. **H** TSA treatment diminished the effect of KD on averaged seizure score. n=7 mice for CD+Vehicle group; n=8 mice for KD+Vehicle group; n=8 mice for CD+TSA group, n=8 mice for KD+TSA group. One-way ANOVA, F_(3,27)_=12.35, P<0.0001. CD+vehicle vs KD+vehicle, *P*=0.0076; CD+TSA vs KD+TSA, *P*=0.9756. *n.s.* indicates not significant. * indicates *p*<0.05; ** indicates *p*<0.01; *** indicates *p*<0.001

Previous studies have indicated that the GABA level was increased in the brain of KD-fed rodent animals [22, 23]. Our recent work reported that KD treatment significantly increased inhibitory synaptic activity in the hippocampus [11]. To determine whether the effects of KD on inhibitory synaptic activity rely on acetylation, we recorded evoked inhibitory postsynaptic currents (eIPSCs) in the hippocampus from CD or KD-fed mice (Fig. 3A). We observed that, while KD increased eIPSC amplitudes in the hippocampal pyramidal neurons compared with those in CD-fed mice, TSA treatment significantly eliminated the difference between CD and KD groups (Fig. 3B, C). Besides, by recording the spontaneous inhibitory postsynaptic currents (sIPSCs), we found that KD dramatically increased the sIPSC frequency. In contrast, TSA treatment elevated sIPSC frequency in the hippocampus from CD-fed mice to a similar level in KD-fed mice (Fig. 3DF). These data indicate that acetylation is required for the effect of KD on inhibitory synaptic activity.

Moreover, we tested whether TSA affects KDs anti-seizure effects. After C57/B6 mice were treated with CD or KD for 3 weeks, KA was then infused into the amygdala to induce seizures the next day after TSA injection (Fig. 3G). We found that, although KD treatment decreased the seizure scores when compared to CD treatment (Fig. 3H), which is consistent with previous reports [11, 24], (TSA injection significantly diminished the difference in seizure activity between CD and KD groups (Fig. 3H). Together, these observations indicate that KD promotes inhibitory synaptic activity and suppresses seizures via histone acetylation elevation.

### Pharmacological inhibition of ErbB4 kinase activity eliminates the effects of KD on inhibitory synaptic activity and seizure resistance

**Fig. 4:**
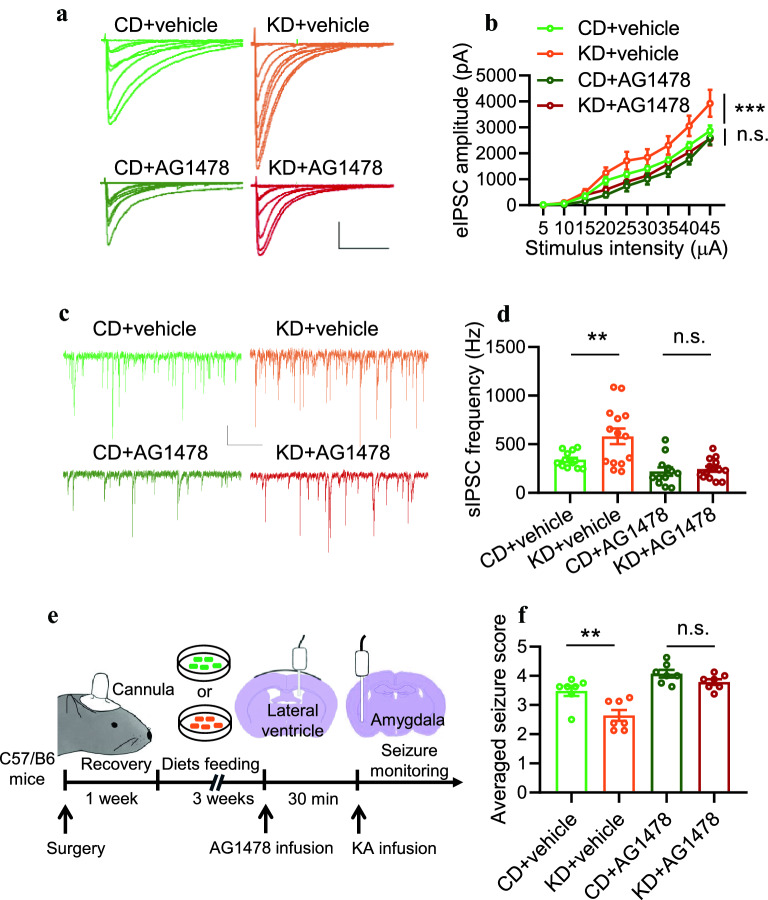
Pharmacological inhibition of ErbB4 kinase activity eliminates the effects of KD on inhibitory synaptic activity and seizure resistance. **A** Representative eIPSC traces from pyramidal neurons in the CA1 region. Scale bars, 100 ms and 1000 pA. **B** AG1478 treatment (5 M) diminished the effect of KD on eIPSC amplitudes. n=18 neurons from 3 mice (CD+vehicle); n=14 neurons from 3 mice (KD+vehicel); n=18 neurons from 3 mice (CD+AG1478); n=14 neurons from 3 mice (KD+AG1478). Two-way ANOVA, CD+Vehicle vs KD+Vehicle, F_(1,267)_=18.68, *P*<0.0001; CD+TSA vs KD+TSA, F_(1,270)_=3.43, *P*=0.0651. **C** Representative sIPSC traces. Scale bars, 2 s and 20 pA. **D** AG1478 treatment diminished the effect of KD on sIPSC frequency. n=13 neurons from 3 mice (CD+vehicle); n=14 neurons from 3 mice (KD+vehicle); n=12 neurons from 3 mice (CD+AG1478); n=14 neurons from 3 mice (KD+AG1478). One-way ANOVA, F_(3,49)_=11.25, *P*<0.0001. CD+vehicle vs KD+vehicle, *P*=0.007; CD+TSA vs KD+TSA, P=0.9996. **E** Schematic of experimental design. After 3 weeks of diet feeding, C57 mice were injected with AG1478 (5 mM, 2 l) into the lateral ventricle. 30 min later, KA was infused to induce seizures. **F** AG1478 treatment diminished the effect of KD on averaged seizure score. n=7 per group. One-way ANOVA, F_(3,24)_=16.46, *P*<0.0001. CD+vehicle vs KD+vehicle, P=0.0041 CD+TSA vs KD+TSA, *P*=0.7313. *n.s.* indicates not significant. * indicates *p*<0.05; ** indicates *p*<0.01; *** indicates *p*<0.001

Our previous work has demonstrated that ErbB4 protein is required for the effects of KD on inhibitory synaptic activity and seizure resistance [11]. It has also been reported that ErbB4 may function independently of its kinase activity [12,14]. To investigate whether ErbB4 kinase activity is critical for the regulation of KD on inhibitory synaptic activity and seizures, we first treated hippocampal slices from CD or KD-fed mice with AG1478, a pharmacological inhibitor of ErbB4 kinase activity [25, 26]. While KD significantly increased eIPSCs amplitudes of pyramidal neurons in the hippocampus compared with the CD group (Fig. 4A, B), these effects were eliminated by AG1478 treatment (Fig. 4A, B). Furthermore, the sIPSC frequency of pyramidal neurons in the hippocampus was elevated by KD treatment. However, AG1478 dramatically decreased the sIPSC frequency in KD-fed mice to a comparable level in CD-fed mice (Fig. 4C, D). These results suggest an essential role of ErbB4 kinase activity in mediating KD effects on inhibitory synaptic transmission.

Next, we utilized the KA-induced seizure model to examine the role of ErbB4 kinase activity in KDs effects on seizures. KA was infused into the amygdala to induce seizures 30 min after AG1478 infusion (Fig. 4E). As shown in Fig. 4F, although KD treatment significantly decreased the seizure score compared with CD, pre-infusion of AG1478 diminished the difference between CD and KD groups. Collectively, these data suggest that EbrB4 kinase activity is critical for the effects of KD on inhibitory synaptic activity and seizure resistance.

### Genetical inhibition of ErbB4 kinase activity eliminates the effects of KD on inhibitory synaptic activity and seizure resistance

**Fig. 5:**
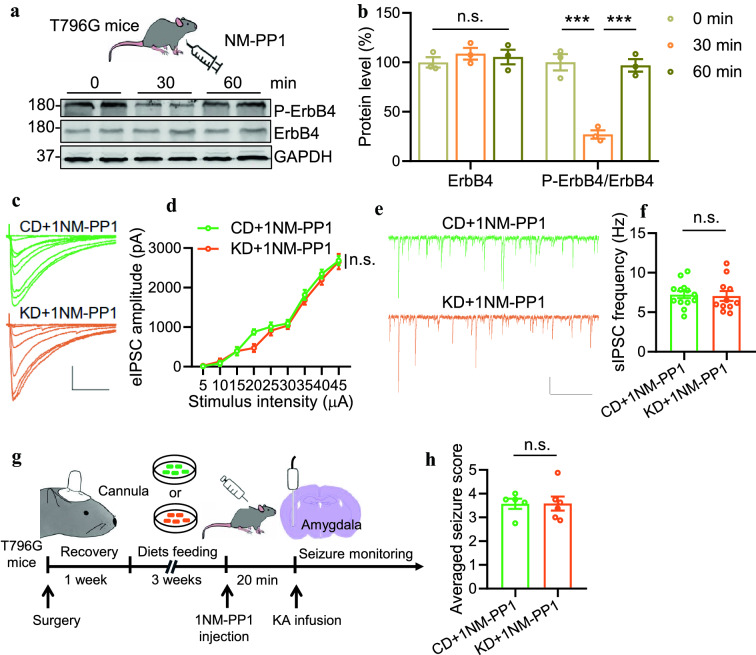
Genetical inhibition of ErbB4 kinase activity eliminates the effects of KD on inhibitory synaptic activity and seizure resistance. **A** Intraperitoneal injection of 1NM-PP1 (0.1 g/g body weight) inhibited ErbB4 phosphorylation in T796G mice in 30 min and recovered in 60 min. **B** Quantitative analysis of data in A. One-way ANOVA, for ErbB4, F_(2,6)_=0.4903, *P*=0.635; for P-ErbB4/ErbB4, F_(2,6)_=70.20, *P*<0.0001. 0 min vs 30 min, *P*=0.0006; 30 min vs 60 min, *P*=0.0008. **C** Representative eIPSC traces from pyramidal neurons in CA1 region. Scale bars, 100 ms and 1000 pA. **D** Comparable amplitudes of eIPSCs in T796G mice fed with KD with those fed with CD after 1NM-PP1 injection. n=13 neurons from 3 mice (CD+1NM-PP1); n=11 neurons from 3 mice (KD+1NM-PP1). Two-way ANOVA, F_(1,198)_=3.237, *P*=0.0735. **E** No difference in the sIPSC frequencies between the two groups. Shown were representative sIPSC traces from pyramidal neurons in the CA1 region. Scale bars, 2 s and 50 pA. **F** Quantitative analysis of data in E. n=13 neurons from 3 mice (CD+1NM-PP1); n=11 neurons from 3 mice (KD+1NM-PP1). Students *t* test, t_(22)_=0.2317, *P*=0.8189. **G** Schematic of experimental design. After three weeks of diet feeding, T796G mice were injected with 1NM-PP1 20 min before KA infusion to induce seizures. **H** Comparable seizure score between the two groups. n=5 T796G mice fed with CD, n=6 T796G mice fed with KD. Students *t* test, t_(9)_=0.02204, *P*=0.9829

Because of the non-selectivity of AG1478 for ErbB4, we could not exclude the possible off-target effects. To address this concern, we employed gene-modified mice-T796G, in which the ATP binding pocket of ErbB4 is enlarged by mutation of the conserved threonine796 to glycine [27]. This line allows us to inhibit ErbB4 specifically using an inhibitor-1NM-PP1. We first verified the inhibitory effect of 1NM-PP1 on the phosphorylation status of ErbB4. Intraperitoneal injection of 1NM-PP1 (0.1 g/g body weight) drastically reduced the P-ErbB4 level in the hippocampus of T796G mice within 30 min. It recovered to its average level after 60 min (Fig. 5A, B). In contrast, vehicle injection exhibited no effects on P-ErbB4 level in T796G mice, and 1NM-PP1 was unable to change P-ErbB4 level in C57/B6 mice (Additional file 1: Fig. S3). Together, these observations suggest an acute, specific and reversible regulation of ErbB4 phosphorylation in T796 mice.

**Fig. 6:**
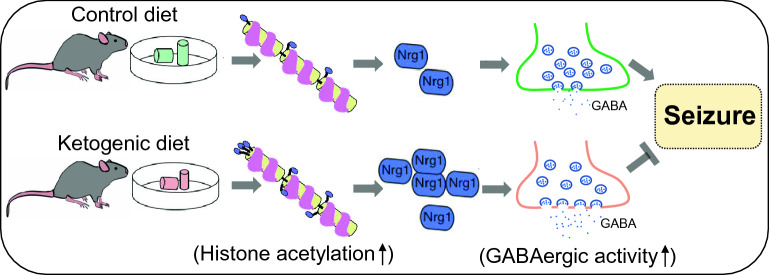
Working model. KD feeding promotes NRG1 expression by elevating histone acetylation, which eventually causes enhanced inhibitory synaptic transmission and anti-seizure effects

After 3 weeks of CD or KD feeding, T796G mice were subject to electrophysiological recordings. We recorded eIPSCs and sIPSCs of pyramidal neurons in the hippocampus with 1NM-PP1 treatment. As shown in Fig. 5C, D, the amplitudes of eIPSCs and the frequency of sIPSCs were comparable between CD and KD groups. These results indicate that specific inhibition of ErbB4 phosphorylation diminishes the impacts of KD on inhibitory synaptic transmission. When we induced seizures in T796G mice by infusing KA into the amygdala 20 min after 1NM-PP1 injection (Fig. 5E), the averaged seizure scores were similar between CD and KD groups (Fig. 4L). Together, these data demonstrate a critical role of ErbB4 kinase activity in mediating the effects of KD on inhibitory synaptic transmission and seizures (Fig. 6).

## Discussion

In the present study, we provide evidence to demonstrate an epigenetic regulation of NRG1 expression by KD and the effects of KD on inhibitory synaptic activity and seizures requires ErbB4 kinase activity. Firstly, KD feeding specifically increased type I NRG1 expression in the hippocampus; Secondly, there was an increase in acetylated histone occupancy at the *Nrg1* promoter locus of KD-fed mice, suggesting an acetylation modulation on *Nrg1* transcription. Indeed, TSA treatment significantly diminished the difference between CD and KD groups; Thirdly, both pharmacological and genetic inhibitions of ErbB4 kinase activity were sufficient to alleviate the effects of KD on inhibitory synaptic activity and seizures. Together, these findings provide insight into the mechanisms underlying KDs beneficial effects and shed light on novel therapeutic interventions for epilepsy.

KD therapy is considered an effective treatment for epilepsy. However, its application in the clinic has largely been restricted due to dietary compliance and adverse side effects [4, 22]. It becomes a pressing need to seek alternative strategies that preserve the therapeutic effects of the KD and avoid its adverse effects. Understanding the cellular and molecular signaling mechanisms underlying the therapeutic effects of the KD is a necessary step to achieve the goal. Previous studies have reported that KD plays a regulatory role in various biological processes, such as mitochondrial respiration, oxidative stress, inflammation, protein post-translational modifications, gut microbiota-brain communication and ion channel properties [28]. These results suggest complex and integrative effects of KD. One potent anti-seizure mechanism is the regulation of KD on inhibitory synaptic activity. A vast amount of evidence has demonstrated that deficiency in GABAergic functions could lead to epilepsy. Various anti-epileptic drugs like benzodiazepines, barbiturates and tiagabine, are known to alleviate seizures by enhancing GABAergic inhibition [29]. In fact, numerous studies have reported that the GABA level was increased in the brains of humans and animal models with KD treatment [22, 23, 30]. In our previous study, we have demonstrated that GABAergic synaptic activity was decreased in the hippocampus of KD-fed mice, which was probably mediated by enhanced NRG1/ErbB4 signaling [11]. The present study extends our previous findings and shows that Type I, but not type II or type III NRG1 expression is increased by KD treatment, suggesting a type-specific regulation of KD on NRG1 expression. In addition, we use both pharmacological and genetic methods to demonstrate that ErbB4 kinase activity is critical for KDs beneficial effects on GABAergic activity and seizures. These results further support a vital role of NRG1/ErbB4 signaling in mediating KDs effects.

It is noteworthy that, it was indicated that overexpression of type I NRG1 in the pyramidal neurons led to reduced glutamatergic transmission in the hippocampus through LIM kinase 1 activation [31, 32], which could be beneficial for the relief of epilepsy. However, in our previous study, we have failed to observe an alteration of glutamatergic transmission in the hippocampus after 3 weeks of KD feeding [11]. One possible reason is that the level of NRG1 is not elevated high enough by 3 weeks of KD feeding to affect excitatory synaptic activity. On the other hand, it is not clear yet at what cell types the NRG1 expression is elevated by KD. A recent study reported that overexpression of NRG1 in GABAergic interneurons caused cortical disinhibition via inhibiting Na_v_ currents [16], which presumably would promote seizure. Thus, it is unlikely that the elevated level of NRG1 in GABAergic interneurons mediates KDs anti-seizure effect. Nevertheless, future study is needed to investigate the potential dose-dependent effects of Nrg1 on excitatory synaptic transmission and at which cell types the KD elevates NRG1 expression occurs.

We also explored the underlying mechanisms through which KD treatment increases NRG1 expression. -hydroxybutyrate, the main form of ketone bodies, is reported to increase histone acetylation and serves as an endogenous HDAC inhibitor [17]. Indeed, several studies have reported the beneficial effects of KD/ketone bodies on oxidative stress and spatial memory, probably through acetylation regulation [33, 34]. On the other hand, previous studies have indicated that dysregulated epigenetic changes, including acetylation of histones, play a prominent role in regulating epileptogenesis [35]. Notably, a well-known anti-epileptic drug-valproic acid (VPA), also an inhibitor of histone deacetylases (HDACs), increases H3 acetylation in the brain [36], eventually suppresses seizures by increasing the levels of GABA in the brain. These findings support the disease-modifying effect of histone acetylation status. By using the CHIP technique, we found increased acetylated histone occupancy at *Nrg1* promoter locus, indicating a direct regulation of acetylation on *Nrg1* transcription. This point was further approved by the fact that the HDAC inhibitor-TSA significantly diminished the effects of KD on GABAergic activity and seizures. Nevertheless, it is not clear how KD-mediated histone acetylation affects NRG1 expression. Future study is warranted to investigate which HDAC is involved in the modulation of KD on acetylation and specific transcription mechanisms that affect type I but not type II or type III *Nrg1* transcription.

Plentiful studies have demonstrated that GABAergic transmission in the brain is tightly regulated by NRG1/ErbB4 signaling [6, 10]. ErbB4 is exclusively expressed in the GABAergic interneurons in the cortex, hippocampus and amygdala [37]. Activation of ErbB4 by its ligand-NRG1 increased the probability of GABA release from the axonal terminal [25, 26, 38]. Our results are in line with previous reports by showing that inhibition of NRG1/ErbB4 signaling decreases GABAergic synaptic activity and thus alleviates KDs effects on seizure control. However, it is still not clear how NRG1/ErbB4 signaling regulates GABA synaptic activity. Previous studies have proposed several potential downstream targets for NRG1/ErbB4 signaling, including the voltage-gated potassium channel Kv1.1, which mediate the effect of NRG1/ErbB4 signaling on the firing of PV^+^ interneurons in the cortex [39]; voltage-gated sodium channel, whose activity in ErbB4^+^ neurons was reduced by NRG1 treatment in vitro [40]; transient outward channel Kv4.2, whose expression was increased by NRG1/ErbB4 signaling through the Akt/mTOR pathway in cultured rat cerebellar granule neurons [41]. Nevertheless, future studies are needed to verify whether such mechanisms underlie the role of NRG1/ErbB4 signaling in the anti-seizure effects of KD.

## Materials and methods

### Reagents and antibodies

Following antibodies were used: Mouse anti-NRG1 (Santa Cruz Biotechnology) (sc-28916; 1:1000 for blotting); Mouse anti-ErbB4 (Santa Cruz Biotechnology) (sc-8050; 1:1000 for blotting); Rabbit anti-P-ErbB4 (Cell Signaling Technology) (Tyr1284; 1:1000 for blotting); Mouse anti-GAPDH (Santa Cruz Biotechnology) (sc-32233; 1:1000 for blotting); Rabbit anti-H3 (Active motive) (61799; 1:1000 for blotting); Rabbit anti-H3k18ac (Active motive) (39587; 1:1000 for blotting); Rabbit anti-H3k27ac (Active motive) (39133; 1:1000 for blotting); Rabbit anti-H4 (Active motive) (61299; 1:1000 for blotting); Rabbit anti-H4k8ac (Active motive) (61103; 1:1000 for blotting); Rabbit anti-H4k12ac (Active motive) (39165; 1:1000 for blotting);); Rabbit anti-acetylated H4 (abcam) (ab233193; 1:200 for CHIP). Rabbit anti-acetylated H3 (Sigma) (06599; 1:200 for CHIP).

Unless otherwise indicated, chemicals were purchased from Sigma-Aldrich. DL-AP5 (0105), CNQX (0190) were purchased from Tocris Bioscience. 1NM-PP1 (HY-13942) and ITSA-1 (HY-100508) were from MedChemExpress.

### Animals

Eight-to twelve-week-old male mice were used for experiments. C57/B6 mice were purchased from the Laboratory Animal Center of Guangdong Province. The primers for genotyping were listed as below: *T796G*, 5- CTT AGC AAT CTG GAC ACA CCAG-3 and 5- CCT ATT GGG AGT GTG TCT GAG TCC CAC TAT CCA GGT TAC G-3 and 5- CCC ACT ATC CAG TTG GTT GGC-3. In all experiments, significant efforts were made to minimize the total number of animals used while maintaining statistically valid group numbers. Animal housing conditions were maintained at a temperature of 221 C, at>30% humidity and a standard 12 h light/dark cycle. All animal experimental protocols were approved by the Animal Ethics Committee of Guangzhou Medical University.

### Diets and feeding

As we reported previously [11], SD (D10070802) (per-calorie macronutrient) contains: 10% protein, 80% carbohydrates, and 10% fat; KD (D10070801) consists of the following: 10% protein and 90% fat. Sources of fat are Soybean oil and cocoa butter. Micronutrient content, fiber, and preservatives are matched according to a per-calorie basis. During experiments, CD or KD was placed in the food well of the cage-top wire lid (stick-like texture). Diets were changed every week.

### Blood ketones and glucose

Blood ketone levels were measured using the blood glucose and ketone monitoring system (FreeStyle Optium Neo, Abbott) according to the manufacturers instructions. Briefly, after sterilization with 70% ethanol, the tail tips of mice under tests were cut using a clean scissor, and a drop of blood was collected. Using the test strip (Abbott), levels of -hydroxybutyrate or glucose were determined.

### Western blot

Brain tissue homogenates were prepared in RIPA Buffer containing (in mM): 50 TrisHCl, pH 7.4, 150 NaCl, 2 EDTA, 1 PMSF, 50 sodium fluoride, 1 sodium vanadate, 1 DTT with 1% sodium deoxycholate, 1% SDS and 1% protease inhibitors cocktails. Samples were resolved on SDS/PAGE and transferred to nitrocellulose membranes, which were incubated in TBS buffer containing 0.1% Tween-20 and 5% milk for 1 h at room temperature before incubating with primary antibodies (overnight at 4 C). After washing, the membranes were incubated with HRP-conjugated secondary antibodies (Absin ImmunoResearch) in the same TBS buffer for 1 h at room temperature. Immunoreactive complex bands were visualized using enhanced chemiluminescence (Pierce) and captured using the Genesys imaging system (Gene Company Limited, UK). Band densities of interested proteins were normalized with by the loading control.

### qRT-PCR analysis

qRT-PCR was performed as described previously [42]. Briefly, total RNA was isolated by using TRIzol reagent (15,596026, Invitrogen). RNA (1 g) was reverse-transcribed with oligo dT-primers using Maxima reverse transcriptase (EP0742, Fermentas) followed by q-PCR with SYBR Green detection (K0222, Fermentas). Samples were assayed in triplicates, with each plate having loading standards in duplicate. RNA levels of different types of *Nrg1* were normalized to those of GAPDH. Primer sequences were: Type I *Nrg1*, 5-AAG GGC AAG AAG AAG GAC CG-3 and 5-AAT CTG GGA GGC AAT GCT GG-3; Type II *Nrg1*, 5-TGC AAG CGG TGC GCA TT-3 and 5-TAC GGT TCA GCT CAT TCC CG-3; Type III *Nrg1*, 5- GCT GTC TGC TTT TCC TCC CTT-3 and 5-TGT TTG TGG CTG AGT TCC TGA-3; GAPDH, 5- GGT TGT CTC CTG CGA CTT CA-3 and 5-CCA CCA CCC TGT TGC TGT AG-3.

### Chromatin immunoprecipitation (CHIP)

The CHIP assay was performed as directed by the manufacturer (Millipore). Briefly, samples were crosslinked with 1% formaldehyde at 37 C for 15 min and then subject to sonication into 2001000 bp fragments. The chromatin was immunoprecipitated with anti-acetylated H3 or H4 primary antibodies. The crosslinking was reversed, and the DNA was isolated on the columns provided by the kit. Real-time PCR was conducted with primers targeted to the promoter of type I *Nrg1* (CAAGAGTAGCCCCGAGACAC and AAAAGTTTGTCCCGGGAGGG). Each experiment was conducted at least three times.

### Electrophysiological recording

Adult male mice were anesthetized with isoflurane. Brains were quickly removed and chilled in ice-cold modified artificial cerebrospinal fluid (ACSF) containing (in mM): 120 Choline-Cl, 2.5 KCl, 7 MgCl_2_, 0.5 CaCl_2_, 1.25 NaH_2_PO_4_, 25 NaHCO_3_, and 10 glucose. Coronal hippocampal slices (300 m) were sectioned in ice-cold modified ACSF using a VT-1000S vibratome (Leica, Germany) and transferred to a storage chamber containing regular ACSF (in mM) (126 NaCl, 3 KCl, 1 MgSO_4_, 2 CaCl_2_, 1.25 NaH_2_PO_4_, 26 NaHCO_3_, and 10 glucose) at 32 C for 30 min and at room temperature (241 C) for additional 1 h before recording. All solutions were saturated with 95% O_2_ / 5% CO_2_ (vol/vol).

Slices were placed in the recording chamber superfused (2 ml/min) with ACSF. Whole-cell patch-clamp recording from CA1 pyramidal neurons was visualized with infrared optics using an upright microscope equipped with an infrared-sensitive CCD camera (DAGE-MTI, IR-1000E). Pipettes were pulled by a micropipette puller (P-97, Sutter instrument) with a resistance of 35 M. Recordings were made with MultiClamp 700B amplifier and 1440A digitizer (Molecular Device).

To record spontaneous inhibitory postsynaptic current (sIPSC), pyramidal neurons were held at -70 mV in the presence of 20 M CNQX and 50 M AP-5, with the pipette solution containing (in mM): 140 CsCl, 10 Hepes, 0.2 EGTA, 1 MgCl_2_, 4 Mg-ATP, 0.3 Na-GTP, 10 phosphocreatine and 5 QX314 (pH 7.40, 285 mOsm). To measure evoked inhibitory postsynaptic currents (eIPSCs), stimulation electrode was positioned on the Schaffer Collaterals (SC)-CA1 pathway,~100 m away from the recording pipette.

In all experiments, series resistance was maintained below 20 M and not compensated. Cells would be rejected if membrane potentials were positive more than -60 mV; or if series resistance fluctuated more than 20% of initial values. Data were filtered at 1 kHz and sampled at 10 kHz.

### Cannula implantation

Adult male mice were maintained anesthetized with isoflurane (23%) and head-fixed in a stereotaxic device (RWD Life Science.Inc). After an incision was made in the scalp, a small hole was drilled into the skull, and a guide cannula (IO: 0.48 mm; RWD Life Science.Inc) was implanted inside the right amygdala (coordinates: anteroposterior, 1.22 mm; dorsoventral, 4.5 mm; mediolateral, 3 mm relative to bregma) or the left lateral ventricle (coordinates: anteroposterior, 0.46 mm; dorsoventral, 2.25 mm; mediolateral, 1.25 mm relative to bregma), and cemented onto the skull with dental cement. Mice were then returned to their homecages for at least 1 week.

### Seizure induction and behavioral monitoring

As previously reported [11], mice with guide cannulas were gently restrained and an infusion cannula (IO: 0.3 mm; RWD Life Science.Inc) was inserted into the amygdala through the guide cannula. 0.15 l of KA (3 mg/ml) was infused into the amygdala at the flow rate of 2 nl/s controlled by microinjector (NanojectIII, Drummond Scientific). The cannula was kept in the right amygdala for two additional min after completion of infusion and withdrew slowly to minimize reflux along the injection tract. Behavioral seizures were classified based on the criteria described by Racine[43] and scored every 5 min by a blinded investigator: stage 0, no seizure; stage 1, arrest and rigid posture; stage 2, head nodding; stage 3, sporadic full-body shaking, spasms; stage 4, chronic full-body spasms; stage 5, jumping, shrieking, falling over; stage 6, violent convulsions or death.

### Experimental design and statistical analysis

Adult male mice (2 months) were used in the present study. Animal or replicate numbers for each experiment and results of the statistical analyses, including degrees of freedom and exact p-values were mentioned in the figure legends. Statistical analyses were performed using GraphPad Prism (GraphPad Software). Students t-test was used to compare data from two groups. One-way ANOVA with sidaks post multiple comparison test was used to compare data from multiple groups. Regular two-way ANOVA with sidaks post multiple comparison test was used in the studies that analyze more than two parameters. All tests were two-sided. All data represent meanSEM, unless otherwise stated. *P*<0.05 was considered to be statistically significant.

## Supplementary Information

**Additional file 1.** Supplementary figures.

## Data Availability

The data of the current study are available from the corresponding author on reasonable request.
