# Supplementary figures and images for: The ketogenic diet increases Neuregulin 1 expression via elevating histone acetylation and its anti-seizure effect requires ErbB4 kinase activity
Source: Cell Biosci. 2021 May 21;11:93. doi: 10.1186/s13578-021-00611-7 (PMC8139023; doi:10.1186/s13578-021-00611-7)

# Supplemental fig. 1

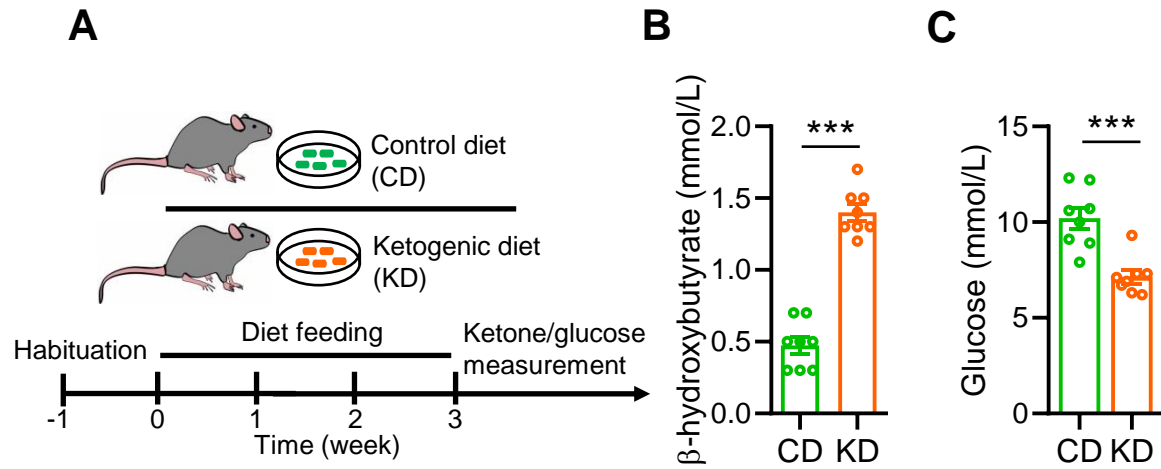

## Supplemental fig. 2

**A**

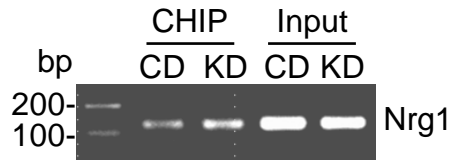

**B**

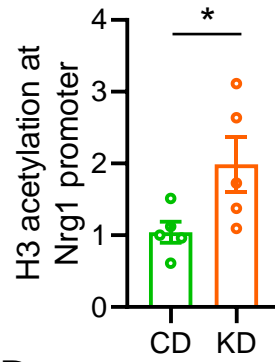

**C**

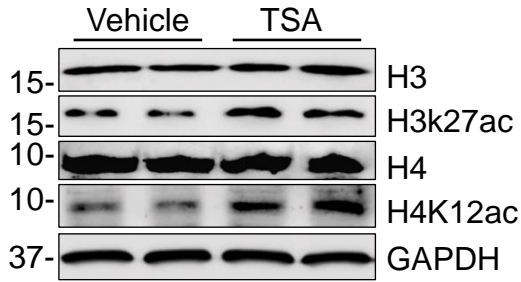

**D**

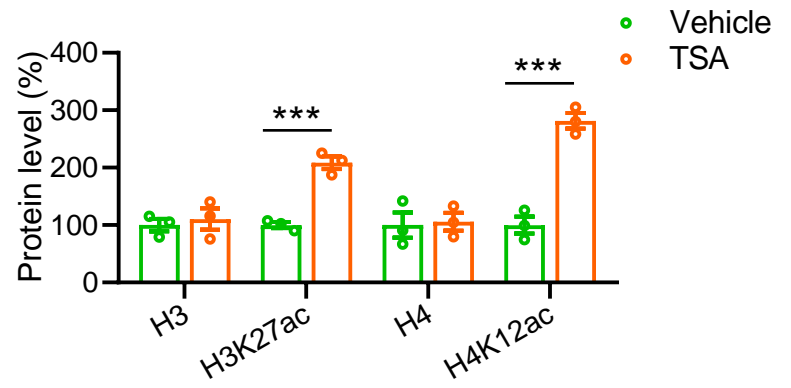

**E**

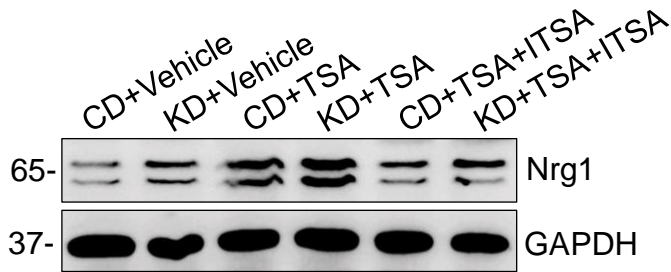

**F**

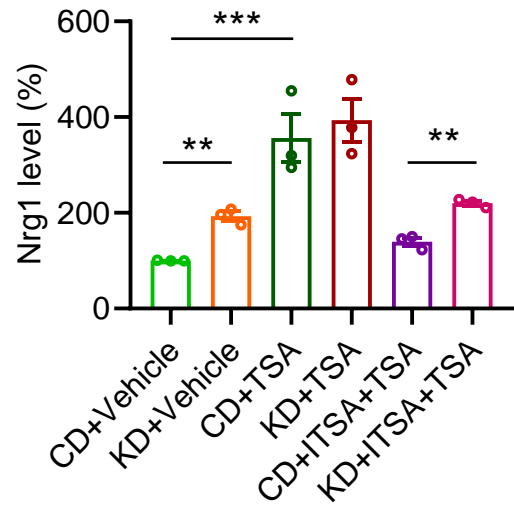

## Supplemental fig. 3

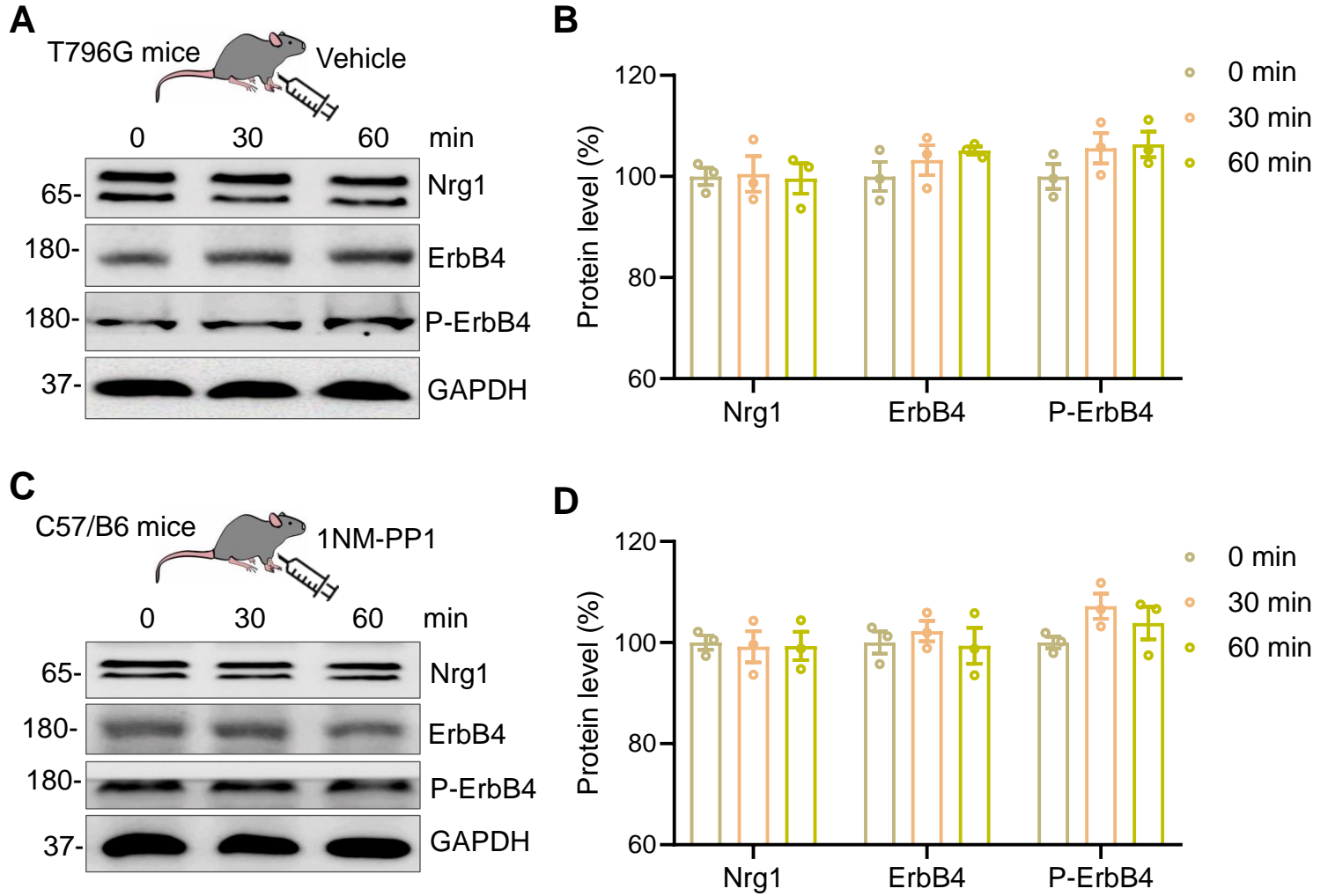

Supplement: Supplementary file 1 — Additional file 1. Supplementary figures. [file 13578_2021_611_MOESM1_ESM.pdf]
